# Visualization and quantitation of the expression of microRNAs and their target genes in neuroblastoma single cells using imaging cytometry

**DOI:** 10.1186/1756-0500-4-517

**Published:** 2011-11-28

**Authors:** Eugene D Ponomarev, Tatiana Veremeyko, Natasha S Barteneva

**Affiliations:** 1 Center for Neurologic Diseases, Brigham and Women's Hospital, Harvard Medical School, Boston, MA, USA; 2 Immune Disease Institute and Program in Cellular and Molecular Medicine, Children's Hospital Boston, Boston, MA, USA; 3 Department of Pediatrics, Harvard Medical School, Boston, MA, USA

**Keywords:** MicroRNA, Target gene, Imaging cytometry, Neuroblastoma, MiR-124, CDK6

## Abstract

**Background:**

MicroRNAs (miRNAs) are regulatory molecules that play an important role in many physiological processes, including cell growth, differentiation, and apoptosis. In addition to modulating normal cellular functions, it has also been reported that miRNAs are involved in the development of many pathologies, including cardiovascular diseases, cancer, inflammation, and neurodegeneration. Methods for the sensitive detection and measurement of specific miRNAs and their cellular targets are essential for both basic research endeavours, as well as diagnostic efforts aimed at understanding the role of miRNAs in disease processes.

**Findings:**

In this study, we describe a novel, imaging cytometry-based protocol that allows for simultaneous visualisation and quantification of miRNAs and their putative targets. We validated this methodology in a neuronal cell line by examining the relationship of the miRNA miR-124 and its known target, cyclin dependent kinase 6 (CDK6). We found that ectopic overexpression of miR-124 resulted in the downregulation of CDK6, decreased cellular proliferation, and induced cellular morphological changes.

**Conclusions:**

This method is suitable for analysing the expression and cellular localisation of miRNAs and target proteins in small cell subsets within a heterogeneous cell suspension. We believe that our cytometry-based methodology will be easily adaptable to miRNA studies in many areas of biomedical research including neuroscience, stem cell biology, immunology, and oncology.

## Background

MiRNAs are small (18-23 nucleotides) non-coding RNAs that regulate the expression of target genes by binding to complementary mRNAs. Binding of an miRNA to its target mRNA leads to either degradation of the mRNA or prevention of its translation [1]. As the study of miRNAs has advanced, it has become evident that these molecules play crucial roles in myriad tissues, including those of the nervous system [1,2]. MiRNAs have been shown to play an important role in cell differentiation, proliferation, and apoptosis [3]. Moreover, deregulation of specific miRNA expression has been connected to several pathologies, including cancer, inflammation, and neurodegenerative disease [4-6].

As interest in the mechanisms and clinical relevance of miRNA-mediated gene regulation increases, there is a demand for new methods that can quantitatively assesses the expression levels of specific miRNAs and their target genes in various subsets of cells. Current methods for measuring the expression of miRNAs include quantitative real-time PCR (qPCR) and in situ hybridisation with specific probes [7,8]. Although qPCR is a sensitive and quantitative method, it does not allow for the measurement of miRNA levels in specific cell subsets within a heterogeneous cell population, nor does it allow for the visualisation of specific miRNA species in particular cellular compartments of sorted cells. Other methods, such as in situ hybridisation, are capable of assessing miRNA expression in specific cell types within tissue sections, but this method is not entirely quantitative, as either semiquantitative western blotting or a recently developed quantitative TaqMan Protein Assay are used to measure target protein levels in the entire cell population rather than in individual cells [9]. Recently described microscopy-based methods allow for the detection of both miRNAs and their targets in tissue sections [10]. However, to our knowledge, no cytometry-based quantitative methods have been described in which the expression of miRNAs and their targets can be measured simultaneously in a single cell. We believe that such methods would be extremely valuable for in vivo studies of miRNA function in a heterogeneous cell population, particularly in the case of clinical specimens. Quantitative imaging cytometry with the ImageStream system allows for quantitative evaluation of internalisation, co-localisation, and trafficking of proteins in various cellular compartments [11-13] and is a method of choice due to its ability to combine morphometric analysis of images with the statistical analysis of a large number of cells (reviewed by Zuba-Surma et al [14].).

The miRNA miR-124 is known to be expressed in the CNS by mature neurons and plays an important role in the differentiation of neuronal progenitors to neurons by targeting several genes, including CDK6 [15,16]. CDK6 is a member of the family of serine-threonine kinases that controls cell cycle progression in many cell types, including neuronal cells [17]. It has been shown that inhibition of CDK6 expression by miR-124 prevents the growth of medulloblastomas that comprise approximately 20% of primary paediatric brain tumors [16].

In this study, we demonstrate that the expression of miR-124 in a neuronal cell line is inversely correlated with expression of CDK6 in individual cells, and that miR-124 overexpression downregulates CDK6, decreases cell proliferation and promotes apoptosis. This was achieved by employing a novel imaging cytometry-based methodological approach that allows for the simultaneous visualisation and quantification of a specific miRNA and its target in individual cells.

## Materials and methods

### Cells

Mouse neuroblastoma NIE115 cells were purchased from ATCC (Manassas, USA) and were maintained in DMEM (ATCC) with 10% FBS (Invitrogen, Carlsbad, USA).

### Antibodies and reagents

A rabbit anti-CDK6 primary antibody (sc-117, used at a dilution of 1:50) was purchased from Santa Cruz Biotechnology (Santa Cruz, USA). The secondary goat anti-rabbit IgG Alexa Fluor 660 conjugated antibody (highly cross- adsorbed, used at a dilution of 1:500) was purchased from Invitrogen (Carlsbad, USA). MiR-124-FITC (cat#38507-04, C = 25 μM), scrambled-miR-FITC (cat#99004-04, C = 25 μM), and miRCURY™ locked nucleic acid (LNA) detection probes were purchased from Exiqon (Woburn, USA) and used at a dilution of 1:100. Hybridisation buffer (25% formamide, 15 mM NaCl and 10% dextran sulphate) was prepared as described previously [8].

### Staining for miR-124 and CDK6

The NIE115 cells were washed with PBS and permeabilised using the BrdU Flow kit (BD Biosciences, San Jose, USA) according to the manufacturer's instructions. After washing with Perm/Wash Buffer from the BrdU Flow kit, the cells were incubated in Perm/Wash buffer containing 50% goat serum for 20 min, after which the anti-CDK6 antibody was added. The cells were incubated with the primary antibody for 30 min at 20°C (room temperature), washed twice with Perm/Wash buffer, and then incubated with the AlexaFluor 660-conjugated secondary antibody in Perm/Wash Buffer at 20°C for 20 min. After incubation, the cells were washed twice in Perm/Wash Buffer and fixed with 1% paraformaldehyde in PBS for 15 min on ice. After fixation, the cells were washed with PBS and resuspended in hybridisation buffer. The cells were incubated for 30 min at 53°C followed by the addition of miR-124-FITC or scrambled-miR-FITC probes. The cells were incubated with the probes for 1 h at 53°C and then washed twice with 1X saline-sodium citrate (SSC) buffer at 53°C, washed once with 0.1× SSC buffer at 53°C, washed once with PBS at 20°C, and finally resuspended in 1% paraformaldehyde in PBS. The cells were stored at 4°C until analysis.

### Transfection with miR-124

5-10 × 10^6 ^neuroblastoma NIE115 cells were transfected with 50 nM of a miR-124a duplex that mimics pre-miR-124a (sense 5'-UAAGGCACGCGGUGAAUGCC-3', antisense: 3'-UUAUUCCGTGCGCCACUUAC-5', Invitrogen, Carlsbad, USA), using Lipofectamine 2000™ (Invitrogen, Carlsbad, USA) according to the manufacturer's instructions. As a negative control for miR-124, control miRNA (Negative Control#1, sense: 5'-AGUACUGCUUACGAUACGGTT-3', antisense: 5'-CCGUAUCGUAAGCAGUACUTT-3', Invitrogen, Carlsbad, USA) was used. Cells were analysed 48 h post-transfection.

### BrdU incorporation assay

Proliferation was assessed by examining bromodeoxyuridine (BrdU) incorporation 14 h after the addition of BrdU to the neuroblastoma cell culture. Analysis of BrdU labelling of cells was performed using the BrdU Flow kit from BD Biosciences (San Jose, USA) according to the manufacturer's instructions.

### Quantitative imaging flow cytometry

After staining for miR-124 and CDK-6 and fixation, cells were adjusted to 10^7 ^cells/ml and transferred to 500 μl siliconised microcentrifuge tubes (Sigma, St Louis, USA) for analysis on an ImageStream 100 imaging cytometer (Amnis Inc, Seattle, USA). We successfully used the AMNIS cytometer in our previous applications to study the extent of transfection of macrophages with microRNA-124 [5]. The ImageStream was equipped with 488, 658, and 405 nm laser sources with variable laser power (from 20 to 200 mW for the 488 nm laser and from 20 to 90 nm for the 658 nm laser) and a brightfield light source. Files of 10,000-20,000 events were acquired for each sample and 200-500 events were acquired for single fluorochrome controls. Single colour controls were collected first to set the optimal laser power and to avoid saturation of the camera. Additional single colour control files were collected in the absence of brightfield illumination for use in creating the compensation matrix with IDEAS software (Amnis, Seattle, USA). The calculated compensation matrix was applied to all files to correct for spectral crosstalk. The resulting compensated cytometry data were further analysed with the IDEAS software program. Gating of cell events with the area and aspect ratio was used to eliminate debris (low area) and multi-cellular events (large area, high aspect ratio) from further analysis, as has been described before [13]. After defining single, focused cells, the identification of appropriate cell subpopulations was done by analysing the cells labelled for BrdU, CDK6, and/or miR-124. The ratio of the fluorescent pixel intensity to the area of the cell in their respective channels was analysed for miR-124 and CDK6 staining using the IDEAS software.

## Results

### Expression of miR-124 is inversely correlated with the expression of CDK6

We initially developed techniques for single staining of CDK6 or miR-124. By employing a matched isotype control antibody, we established that preliminary blocking of permeabilised samples with 50% goat serum eliminated non-specific binding of the anti-CDK6 primary antibody, but did not perturb our ability to sensitively detect CDK6 protein in the cells, as shown in Additional file 1: Figure S1a. For miR-124 detection, we adapted a histological method for in situ hybridisation to the analysis of the cell suspension, and adjusted the temperature of hybridisation and the concentration of the probe. As a control for non-specific binding of the miR-124-FITC LNA probe, we used the scrambled-miR-FITC LNA probe from the same company (Exiqon). We found that at a dilution of 1:100 and at a hybridisation temperature of 53°C, binding of the non-specific scrambled-miR-FITC LNA probe (SCR probe) was practically undetectable, while binding of the miR-124-FITC LNA probe (miR-124 probe) was easily detectable (Additional file 1: Figure S1b).

**Figure 1:**
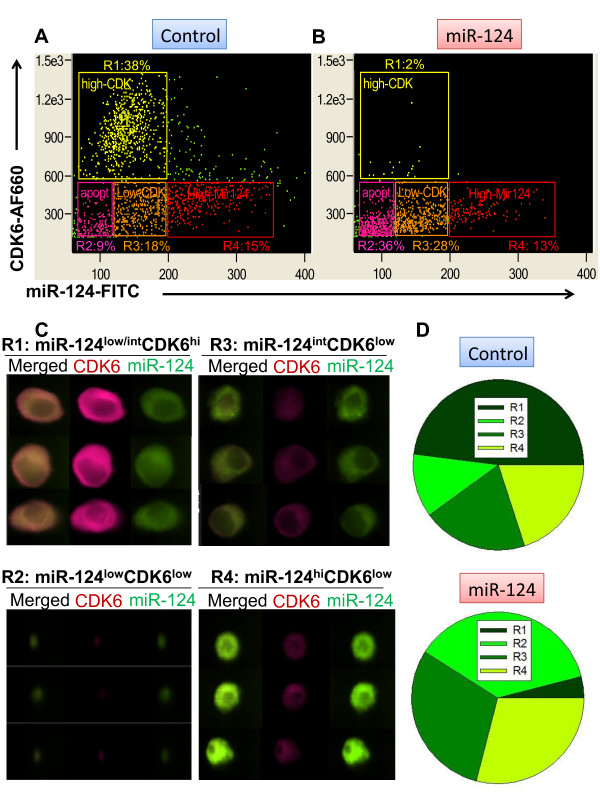
**Analysis of the expression of miR-124 and CDK6 in neuroblastoma cells transfected with control miRNA (a) and miR-124 (b)**. The cells were transfected and stained for miR-124 and CDK6 as described in *Materials and Methods*. The relative fluorescence intensity level for miR-124-FITC (x-axes) vs. CDK6-AF660 (y-axes) is shown in (**a**) and (**b**). Representative images of cells from "control" cell subpopulations-miR-124^low/int^CDK6^hi ^(gate R1); miR-124^low^CDK6^low^(gate R2); miR-124^int^CDK6^low ^(gate R3); and miR-124^hi^CDK6^low ^(gate R4)-are shown in (**c**). The data is representative of four separate experiments. The average percentages for subsets from gates R1-R4 is shown in **(d)**. In (**c**) the expression of miR-124 is shown in green and the expression of CDK6 is shown in red. Merged images show the expression of both CDK6 and miR-124. Three representative images are shown for each subset

A critical component of our desired methodology was the combined detection of CDK6 and miR-124 in single cells. The hybridisation conditions for the miR-124 probe included the presence of formamide in the hybridisation buffer and high temperature (53°C) which likely resulted in the denaturation of CDK6 and substantially decreased antibody staining (data not shown). To avoid this complication, we performed staining for CDK6 and fixation, followed by hybridisation with the miR-124 probe as a sequential step. Our approach is different from previously published methods for miRNA analysis in tissue sections where staining with antibodies for a target protein was performed after hybridisation with a probe for miRNA [17]. We found that our approach produced bright staining for CDK6 before and after hybridisation conditions (Additional file 1: Figure S1c). We also compared the fluorescence intensity of miR-124 expression in control cells or cells transfected with miR-124 using a FITC-labelled probe for miR-124. We found that miR-124 transfected cells became smaller in size and had a comparable level of overall fluorescence for the miR-124-FITC probe, but most of the cytoplasmic areas were brighter in miR-124 transfected cells, as shown in Additional file 1: Figure S1d. We also selected two fluorophores with practically no spectral overlap on the ImageStream 100 cytometer (FITC and Alexa Fluor 660), which eliminated spectral compensation issues. Finally, we measured the expression of miR-124 and CDK6 in an experimental setup typically used in the field of miRNA analysis; thus, we compared the level of expression of both molecules in cells transfected with control miRNA or miR-124. The relative level of expression was evaluated by the ratio between two parameters: fluorescence intensity and the area of fluorescent signal in respective channels. We found that in the control cells there was a distinct distribution pattern for the expression of miR-124 and CDK6, resulting in four distinguishable subsets: cells that expressed high levels of CDK6 and low or intermediate levels of miR-124 (Figure 1a, gate R1), cells that expressed low levels of CDK6 and low levels of miR-124 (Figure 1a, gate R2), cells that expressed low levels of CDK6 and intermediate levels of miR-124 (Figure 1a, gate R3), and cells that expressed low levels of CDK6 and high levels of miR-124 (Figure 1a, gate R4). Representative images of cells from each region are shown in Figure 1c. In the R1 region cells (miR-124^low/int^CDK6^hi^), miR-124 was located primarily in the cytoplasm, which is consistent with the known pattern of miRNA localisation. R2 region cells (miR-124^low^CDK6^low^) were likely apoptotic, as these cells had all the morphological characteristics of cells undergoing apoptosis such as small size, absent nuclei, and low expression levels of both miR-124 and CDK6. The cells from regions R3 (miR-124^int^CDK6^low^) and R4 (miR-124^hi^CDK6^low^) had intermediate and high levels of miR-124 expression, respectively, and low levels of CDK6. Thus, our analysis demonstrated that in a population of neuroblastoma cells, there is an inverse correlation between miR-124 expression and CDK6 expression in individual cells: cells with high levels of CDK6 had low or intermediate levels of miR-124 expression (Figure 1a, gate R1), whereas most of the cells with intermediate and high levels of miR-124 expressed low levels of CDK6 (Figure 1a, gate R3-R4). We found a few cells that were miR-124^hi^CDK6^hi^, shown in Figure 1a in the upper right quadrant as green dots. These cells represented dividing cells (Additional file 2: Figure S2a) or cell doublets consisting of two cells, one of which was CDK6^hi ^while the other was miR-124^hi ^(Additional file 2: Figure S2b). These studies demonstrate the utility of combined cytometry-based miRNA and target detection. It is critical to note that several parameters should be optimised in performing this method. Precise incubation times and temperature control are crucial for obtaining reproducible data, and single-colour control samples are necessary to exclude saturation events and to optimise laser power. In addition, the use of fluorochromes that minimise spectral overlap will help ensure that miRNA and target detection is sensitive and robust.

Overexpression of miR-124 resulted in the downregulation of CDK6. When we compared the level of expression of CDK6 in "control" cells versus cells transfected with miR-124, we found that the CDK6^hi ^population was significantly reduced (from 38 to 2% for gate R1) (Figure 1a, b). This suggests that overexpression of miR-124 resulted in downregulation of CDK6 in the entire population of the cells. We found that the number of cells with a small size and low levels of miR-124 and CDK6 was substantially increased (from 9 to 36%) (Figure 1a, b; gate R2). We speculated that the increase in the number of miR-124^low^CDK6^low ^cells in gate R2 upon miR-124 overexpression was due to enhanced apoptosis. In support of this hypothesis, we found that these cells had all the morphological characteristics of cells undergoing apoptosis (Additional file 3: Figure S3, R2: miR-124^low^CDK6^low^). Representative images of cells from the other regions after transfection with miR-124 are shown in Additional file 3: Figure S3. As is readily apparent, miR-124-transfected cells were smaller in comparison to "control" cells, and contained relatively high levels of miR-124 and low levels of CDK6 (Additional file 1 Figure S1, R3-R4). The average percentage of cells in regions R1-R4 for control cells vs. cells transfected with miR-124 is summarised in Figure 1d. Thus, our cytometry-based method was able to effectively demonstrate that overexpression of miR-124 results in decreased expression of CDK6 in a neuronal cell line.

### Overexpression of miR-124 inhibits cellular proliferation

**Figure 2:**
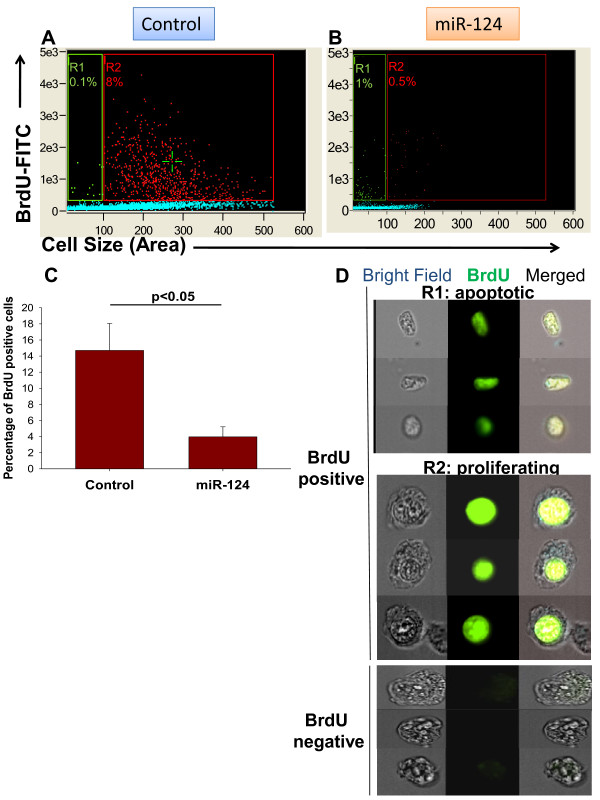
**Comparison of the level of proliferation of neuroblastoma cells transfected with control miRNA (a) and miR-124 (b)**. The cells were transfected and proliferation was assessed by the BrdU incorporation assay as described in *Materials and Methods*. The cell size (area of the cell) and the BrdU-FITC fluorescence intensity are shown along the x-axis and y-axis, respectively, in (**a**) and (**b**). There representative experiment is shown in (**a **and **b**) and mean ± S.E. of three separate experiments is shown in **(c)**. Representative images of BrdU^- ^(negative) and BrdU^+ ^(positive) cells of small size (gate R1) and large size (gate R2) are shown in (**c**). In (**c**) the expression of BrdU is shown in green and the bright field image of the cells is shown in grey. Three representative images are shown for each subset. **In (d)**, the bright field images of the cells are shown in grey (left images), the expression of BrdU is shown in green (middle images) and merged images are shown on the right. Three representative images are shown for each subset

Since CDK6 is a well-characterised mediator of cell cycle progression, we compared the proliferation of cells transfected with control miRNA versus cells transfected with miR-124 by employing the BrdU incorporation assay. We found that overexpression of miR-124 decreased the percentage of BrdU^+ ^cells 14 h post-BrdU treatment by 5.4-fold (from 8.1 to 1.5%), as shown in a representative experiment in Figure 2b (gates R1 and R2). Interestingly, the "control" BrdU^+ ^cells were larger in size (Figure 2a, gate R2) and showed nuclear BrdU localisation (Figure 2c; BrdU^+^, gate R2), while most of the BrdU^+ ^cells in the miR-124-transfected population were smaller in size (Figure 2d, gate R1) and exhibited BrdU throughout the cytoplasm (Figure 2d; BrdU^+^, gate R1). This suggests that most of the BrdU^+ ^cells in the miR-124-transfected population were apoptotic, as the nuclear membrane disappears during apoptosis. This is consistent with previous results that demonstrated that transfection of NIE115 cells with miR-124 results in an increase in the number of cells that have an apoptotic morphology (Figure 1a-d; gate R2). Thus, transfection of neuroblastoma cells with miR-124 results in a decrease in cell proliferation and an increase in apoptosis, which is in concordance with the downregulation of CDK6 by miR-124.

## Discussion

We have demonstrated that the imaging cytometry approach described in this report can be used for sensitive and quantitative evaluation of miRNA and target expression in individual cells. This method may also be useful for the validation of predicted target genes. The use of a fluorescently-labelled miRNA probe in combination with a fluorochrome-conjugated antibody to the miRNA-targeted gene product and excitation from different lasers (488 nm and 658 nm, respectively) made it possible to provide good signal separation with little effect on the overall fluorescent signal. Important features of this technique include i) its feasibility for the analysis of very small numbers of cells and ii) its suitability for detailed characterisation of miRNA and target expression in diverse cell subsets through the use of fluorochrome-conjugated antibodies specific for surface markers. This methodology will be particularly useful for the analysis of miRNA expression/function in immune and stem cells, as well as cells obtained from solid tissues and tumours. We did not experience any methodological difficulties or reduced effectiveness of the method for primary isolated cells when compared to cultured cells, since all the procedures, including cell fixation, permeabilisation, staining with antibodies, and the process of hybridisation with the miRNA-specific probes are virtually identical for primary isolated and cultured cells.

Our new imaging cytometry-based approach has several other advantages over currently used methods for the quantification of miRNA expression and its putative targets. Use of qPCR or proteomics-based assays presume the destruction of the tissue under examination and, even if these assays are combined with FACS (including single-cell qPCR and sensitive mass-spectrometry analysis), these methods do not allow for simultaneous measurement of specific miRNA and/or target levels and the analysis of miRNA and target protein distribution in cellular compartments. By using this imaging cytometer-based protocol, part of the processed sample can be used for quantification by qPCR and/or parallel analysis by conventional flow cytometry.

There has recently been much progress in microscopy-based methods combined with multicolour imaging for the detection of miRNA and its targets [10,18]. These new methods allow for simultaneous analysis of miRNA and putative target expression in tissue sections. Special software enables per-cell analysis that can be displayed as a scatter plot in a manner similar to the presentation of flow cytometry data [10]. Although these methods can be used for the analysis of cells in tissues that cannot be isolated as cell suspensions (e.g. adult brain), they also have several shortcomings. First, microscopy-based methods preclude the analysis of statistically powerful cell numbers, as at most several hundred cells can be realistically analysed in comparison to tens or even hundreds of thousands of events with flow and imaging cytometry. Secondly, staining of tissue sections regularly conjures artefacts that negatively affect immunofluorescence analysis, making it difficult to either use certain antibodies to measure the expression of the target protein or to identify particular cell types [19]. Third, the flow imaging cytometer has all the flexibility of a regular flow cytometer, allowing problems connected with signal amplification and spectral compensation to be overcome, and facilitating the detection of even weak fluorescent signals. Although we analysed our samples on an imaging cytometer, a conventional flow cytometer is perfectly suitable for most of the data collection and analysis, allowing the methodology outlined here to be employed by many scientists who only have access to standard FACS equipment.

In conclusion, we believe that the method described in this manuscript will be of great value for functional studies of the roles miRNAs play in many areas of biology and biomedicine, including the immune response, cancer development, stem cell generation, cell growth and differentiation, apoptosis, and neurogenesis.

## List of abbreviations

BrdU: Bromdeoxyuridine; CDK: Cycline dependent kinase; CNS: Central nervous system; DMEM: Dulbecco's modified eagle medium; FBS: Fetal bovine serum; FITC: Fluorescein isothiocyanate; miRNA: MicroRNA; PBS: Phosphate-buffered saline; qPCR: Quantitative real-time polymerase chain reaction; SSC: Saline sodium citrate.

## Conflict of interests

The authors declare that they have no competing interests.

## Authors' contributions

EDP and TV designed the research, performed the experiments, analyzed the data and drafted paper. NSB and EDP coordinated research, performed imaging cytometry analysis, wrote the manuscript and provided finding. All authors read and approved the final manuscript.

## Supplementary Material

Addtional file 1 **Single stain controls for detection of CDK6 and miR-124**. (A) Neuroblastoma cells were fixed, permeabilized, blocked with 50% goat serum and stained with anti-CDK6 or Isotype control as described in *Materials and methods*. The histograms with anti-CDK6 staining are shown on the top and staining for with isotype control is shown on the bottom. (B) Neuroblastoma cells were fixed, permeabilized and then were subjected to hybridization with miR-124-FITC LNA (miR-124 probe, top histogram) or scrambled-miR-FITC LNA (SCR probe, bottom histogram) probes as described in *Materials and Methods*. (C) Representative images of the cells stained with anti-CDK6 antibodies before (left) and after (right) the cells were subjected co conditions for hybridization with LNA probes are shown. (D) Comparison of cell morphology and miR-124 expression in the cells that were transfected with Control miRNA (left) or miR-124 (right). The cells were transfected as described in *Materials and methods *and then cells were subjected to hybridization with miR-124-FITC LNA probe. Click here for file

Addtional file 2 **Characterization of minor population of miR-124^hi^CDK6^hi ^cells**. Representative images of miR-124^hi^CDK6^hi ^subset of control cells (indicated as green dots in upper right quadrant of Figure 1A) are shown. Most of the events in miR-124^hi^CDK6^hi ^subset represent cell doublets either as dividing cells (A) or cell aggregates (B). Click here for file

Addtional file 3 **Representative images of miR-124-transfected cells from miR-124^low^CDK6^low ^(gate R2), miR-124^int^CDK6^low ^(gate R3) and miR-124^hi^CDK6^low ^(gate R4) subsets are shown**. The cells were transfected with miR-124 and 48 hours later were stained for miR-124 and CDK6 as described in *Materials and Methods*. Staining for miR-124 is shown in green and staining for CDK6 is shown in red. Merged images show staining for both CDK6 and miR-124. Three representative images are shown for each subset. Click here for file
