# Supplementary figures and images for: Visualization and quantitation of the expression of microRNAs and their target genes in neuroblastoma single cells using imaging cytometry
Source: BMC Res Notes. 2011 Nov 28;4:517. doi: 10.1186/1756-0500-4-517 (PMC3250958; doi:10.1186/1756-0500-4-517)

**A**

**Anti-CDK6**

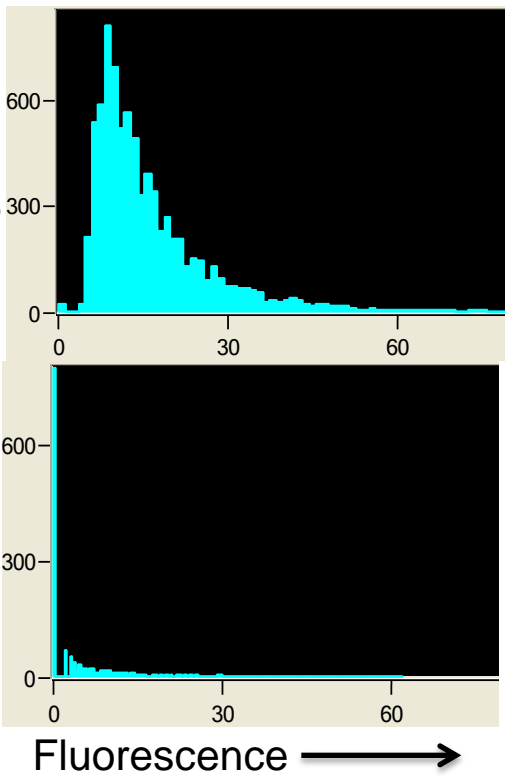

**B**

**miR-124 probe**

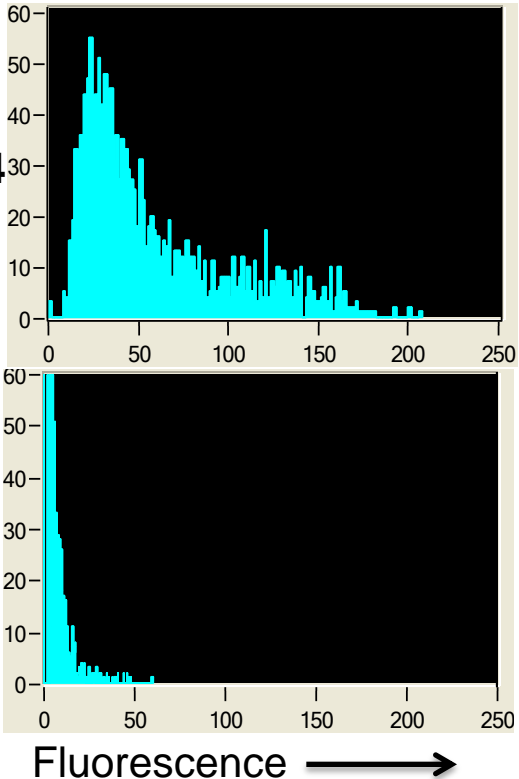

**C**

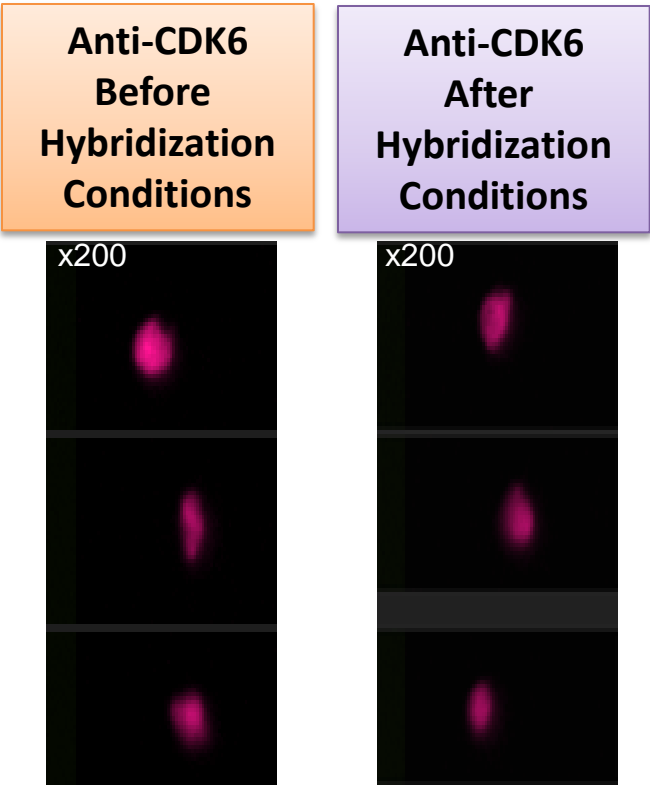

**D**

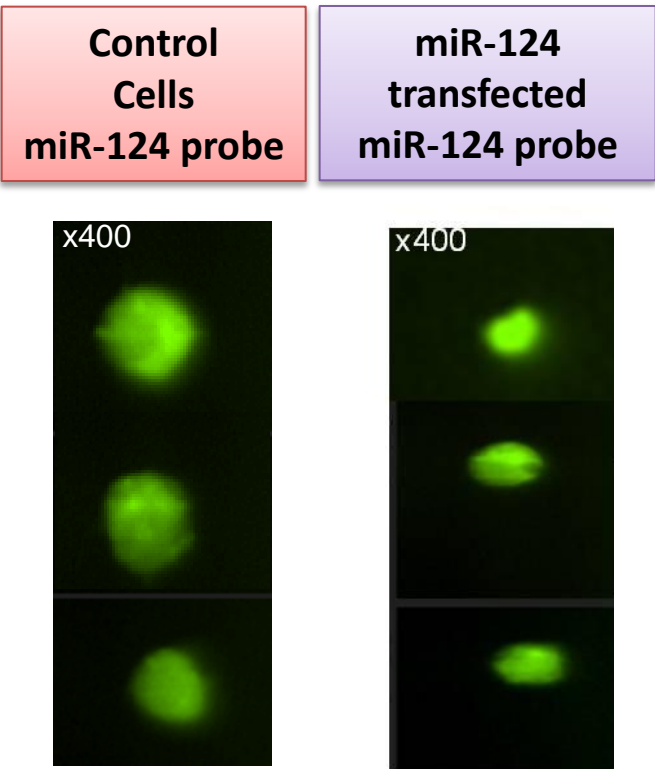

Supplement: Addtional file 1 — Single stain controls for detection of CDK6 and miR-124. (A) Neuroblastoma cells were fixed, permeabilized, blocked with 50% goat serum and stained with anti-CDK6 or Isotype control as described in Materials and methods. The histograms with anti-CDK6 staining are shown on the top and staining for with isotype control is shown on the bottom. (B) Neuroblastoma cells were fixed, permeabilized and then were subjected to hybridization with miR-124-FITC LNA (miR-124 probe, top histogram) or scrambled-miR-FITC LNA (SCR probe, bottom histogram) probes as described in Materials and Methods. (C) Representative images of the cells stained with anti-CDK6 antibodies before (left) and after (right) the cells were subjected co conditions for hybridization with LNA probes are shown. (D) Comparison of cell morphology and miR-124 expression in the cells that were transfected with Control miRNA (left) or miR-124 (right). The cells were transfected as described in Materials and methods and then cells were subjected to hybridization with miR-124-FITC LNA probe. [file 1756-0500-4-517-S1.PDF]

**A**

Merged

miR-124

CDK6

Bright Field

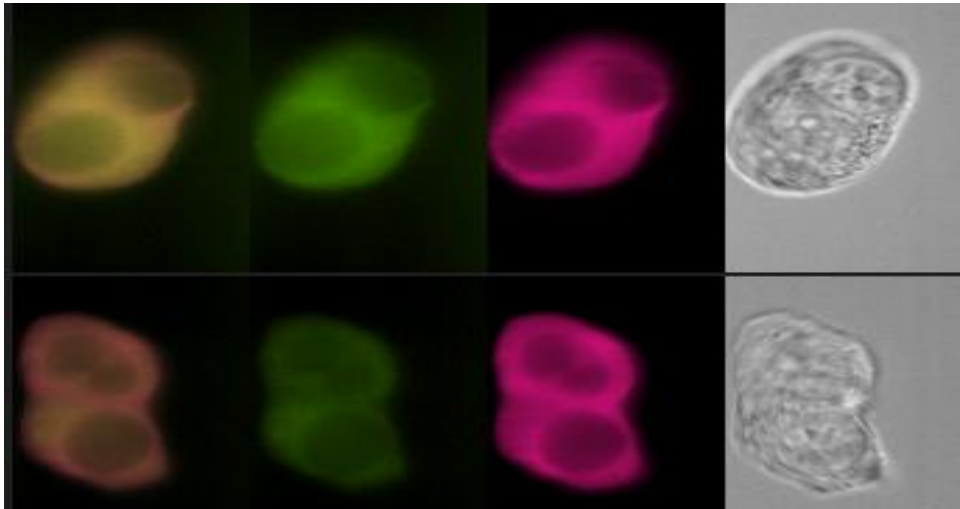**B**

Merged

miR-124

CDK6

Bright Field

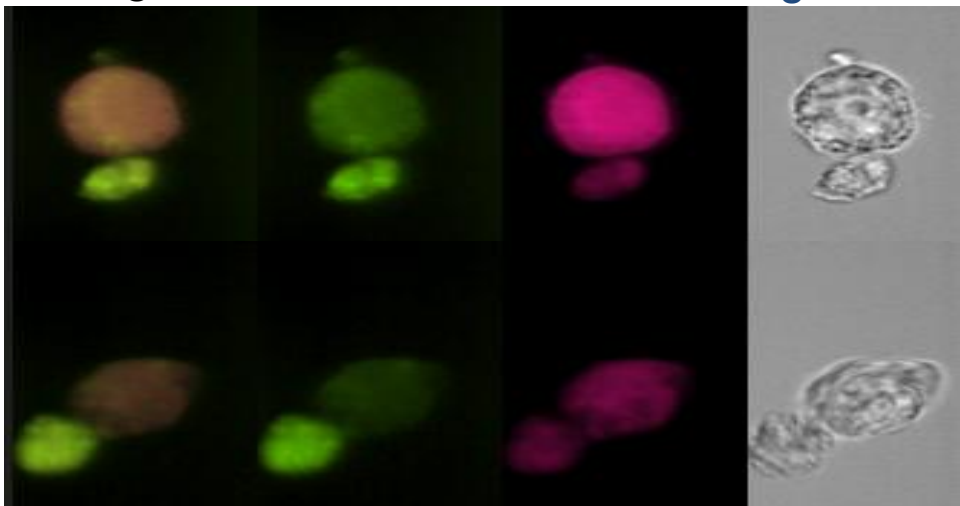

Supplement: Addtional file 2 — Characterization of minor population of miR-124hiCDK6hi cells. Representative images of miR-124hiCDK6hi subset of control cells (indicated as green dots in upper right quadrant of Figure 1A) are shown. Most of the events in miR-124hiCDK6hi subset represent cell doublets either as dividing cells (A) or cell aggregates (B). [file 1756-0500-4-517-S2.PDF]
