# Supplementary material for: Visualization and quantitation of the expression of microRNAs and their target genes in neuroblastoma single cells using imaging cytometry
Source: BMC Res Notes. 2011 Nov 28;4:517. doi: 10.1186/1756-0500-4-517 (PMC3250958; doi:10.1186/1756-0500-4-517)
Supplement: Addtional file 3 — Representative images of miR-124-transfected cells from miR-124lowCDK6low (gate R2), miR-124intCDK6low (gate R3) and miR-124hiCDK6low (gate R4) subsets are shown. The cells were transfected with miR-124 and 48 hours later were stained for miR-124 and CDK6 as described in Materials and Methods. Staining for miR-124 is shown in green and staining for CDK6 is shown in red. Merged images show staining for both CDK6 and miR-124. Three representative images are shown for each subset. [file 1756-0500-4-517-S3.PDF]

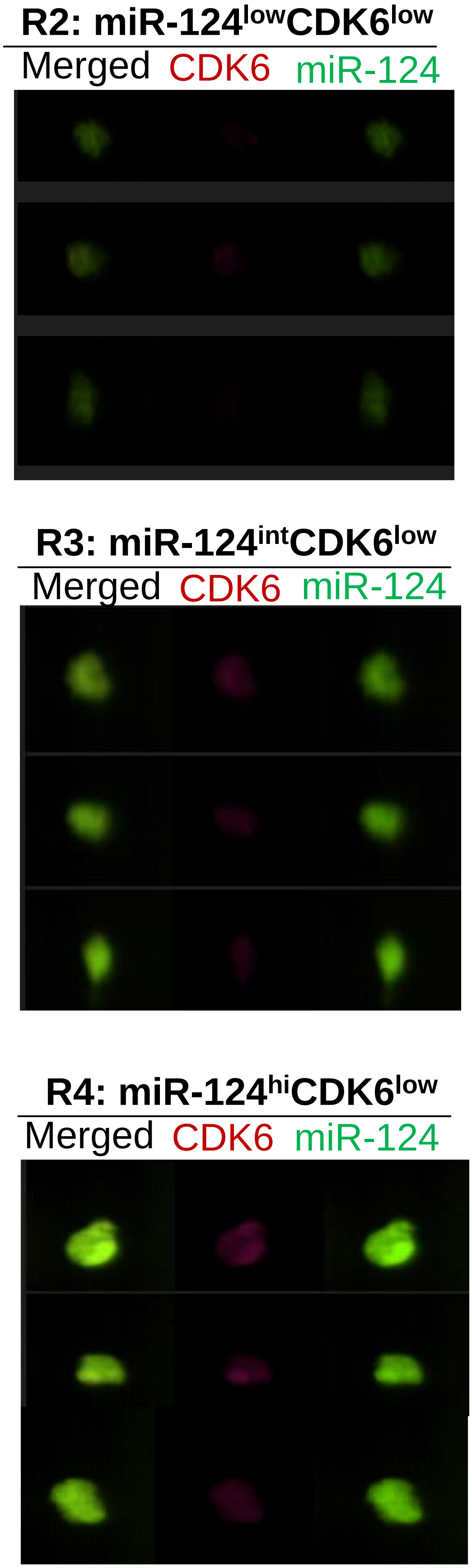
R2: miR-124lowCDK6low
Merged
CDK6
miR-124
R3: miR-124intCDK6low
Merged
CDK6
miR-124
R4: miR-124hiCDK6low
Merged
CDK6
miR-124
